# MicroRNA160 Modulates Plant Development and Heat Shock Protein Gene Expression to Mediate Heat Tolerance in *Arabidopsis*

**DOI:** 10.3389/fpls.2018.00068

**Published:** 2018-02-01

**Authors:** Jeng-Shane Lin, Chia-Chia Kuo, I-Chu Yang, Wei-An Tsai, Yu-Hsing Shen, Chih-Ching Lin, Yi-Chen Liang, Yu-Chi Li, Yun-Wei Kuo, Yu-Chi King, Hsi-Mei Lai, Shih-Tong Jeng

**Affiliations:** ^1^Department of Life Sciences, National Chung Hsing University, Taichung, Taiwan; ^2^Department of Life Science, Institute of Plant Biology, National Taiwan University, Taipei, Taiwan; ^3^Department of Crop Environment, Hualien District Agricultural Research and Extension Station, Council of Agriculture, Hualien, Taiwan; ^4^Institute of Plant and Microbial Biology, Academia Sinica, Taipei, Taiwan; ^5^Department of Agricultural Chemistry, National Taiwan University, Taipei, Taiwan

**Keywords:** Arabidopsis, heat stress, miR160, *ARF10*, *ARF16*, *ARF17*

## Abstract

Global warming is causing a negative impact on plant growth and adversely impacts on crop yield. MicroRNAs (miRNAs) are critical in regulating the expression of genes involved in plant development as well as defense responses. The effects of miRNAs on heat-stressed *Arabidopsis* warrants further investigation. Heat stress increased the expression of miR160 and its precursors but considerably reduced that of its targets, *ARF10, ARF16*, and *ARF17*. To study the roles of miR160 during heat stress, transgenic *Arabidopsis* plants overexpressing *miR160 precursor* a (160OE) and artificial miR160 (MIM160), which mimics an inhibitor of miR160, were created. T-DNA insertion mutants of miR160 targets were also used to examine their tolerances to heat stress. Results presented that overexpressing miR160 improved seed germination and seedling survival under heat stress. The lengths of hypocotyl elongation and rachis were also longer in 160OE than the wild-type (WT) plants under heat stress. Interestingly, MIM160 plants showed worse adaption to heat. In addition, *arf10, arf16*, and *arf17* mutants presented similar phenotypes to 160OE under heat stress to advance abilities of thermotolerance. Moreover, transcriptome and qRT-PCR analyses revealed that *HSP17.6A, HSP17.6II, HSP21*, and *HSP70B* expression levels were regulated by heat in 160OE, MIM160, *arf10, arf16*, and *arf17* plants. Hence, miR160 altered the expression of the heat shock proteins and plant development to allow plants to survive heat stress.

## Introduction

Plants are unable to escape from the various environmental stresses via moving. These stresses, for instance, salt, drought, and heat, seriously affect plant growth, development, and even crop yields. Heat stress is defined as an increase of 5°C or more than the optimal temperature (Guan et al., 2013). Crop yield is highly sensitive to temperature. Since 1977, the productivities of rice (*Oryza sativa*) and wheat (*Triticum aestivum*) have steadily reduced due to heat stress (Long and Ort, 2010). Hence, research on the heat tolerance of plants is valuable. Heat stress causes an excessive increase in membrane fluidity, a disruption of protein function and turnover, and metabolic imbalances in plants (Moreno and Orellana, 2011). These abnormal cellular processes could severely affect the plant and ultimately cause death. Hence, plants have developed various mechanisms to regulate and enhance their heat tolerance. Heat shock proteins (HSPs) and heat stress transcription factors (HSFs) are considered to be the central components of responses to heat stress in plants (Nover and Scharf, 1997; Kotak et al., 2007). HSFs recognize heat stress elements (HSE, 5′-GAAnnTTC-3′) and induce the expression of several heat stress-related genes, including HSPs (Busch et al., 2005). HSPs are divided into five major classes, namely, HSP100, HSP90, HSP70, HSP60, and small heat shock proteins (sHSPs) (Iba, 2002). HSPs are involved in either or both the maintenance and restoration of protein homeostasis upon heat stress (Kotak et al., 2007). HSP101, HSP70, HSP17.6, and HSP17.7 can protect plant cells from heat-induced programmed cell death (Rikhvanov et al., 2007). HSP21 is notable for maintaining plastid-encoded RNA polymerase-dependent transcription in chloroplast development under heat stress (Zhong L. et al., 2013).

Plant hormones also participate in the regulation of heat responses. The addition of abscisic acid (ABA), ethylene, and salicylic acid can protect plants from heat-induced oxidative damage (Larkindale and Knight, 2002). Under heat stress, ABA induces hydrogen peroxide (H_2_O_2_) production through respiratory burst oxidase protein D (RbohD) and RbohF, to regulate heat-related downstream genes (Larkindale et al., 2005). In barley and *Arabidopsis*, high temperature represses the expression of *YUCCA* auxin biosynthesis genes (Oshino et al., 2007; Sakata et al., 2010). The reduction of auxin results in male sterility and further affects fruit setting rate. The addition of auxin can restore the male sterility caused by heat stress (Oshino et al., 2011). In *Arabidopsis*, high temperature also affects intracellular auxin homeostasis (Hanzawa et al., 2013) and promotes auxin-mediated hypocotyl elongation (Gray et al., 1998). The auxin-related microRNAs (miRNAs) and auxin response transcription factors (ARFs) regulate the auxin signaling pathway in plant thermotolerance (Kruszka et al., 2014).

Small RNAs (sRNAs) are 21–24 nucleotide noncoding RNAs with crucial regulatory roles in various responses of plants (Bartel, 2004). Small interfering RNAs (siRNAs) and miRNAs are two major classes of sRNAs (Mallory and Vaucheret, 2006). The Dicer-like (DCL) ribonuclease family is involved in the biogenesis of sRNAs (Tang et al., 2003; Kurihara and Watanabe, 2004). The RNA-induced silencing complexes incorporated with sRNAs can recognize targets of sRNAs and cause target genes to silence post-transcriptionally (Martinez et al., 2002; Bartel, 2004).

sRNAs play multiple roles in plant development and nutrient homeostasis (Bartel, 2004; Huijser and Schmid, 2011; Meng et al., 2011). During embryogenesis, miR160 activates ARFs that modulate expression of early auxin-responsive genes (Liu et al., 2007, 2010). *ARF10* and *ARF16*, two of miR160 targets, regulate *ABI3* expression to induce seed dormancy (Liu et al., 2013). Several sRNAs have also been identified during plant stress responses, such as wounding (Lin et al., 2012, 2013), drought (Li et al., 2008; Ni et al., 2013), salt (Feng et al., 2013; Ni et al., 2013), and heat (Yan et al., 2012; Guan et al., 2013; Li et al., 2014; Stief et al., 2014). Microarray analysis identified *ARF16*, one of the miR160 targets, was repressed by heat, in *Arabidopsis* (Li et al., 2014). High-throughput sequencing revealed 32 miRNA families, among them, miR156, miR160, and miR172, which might be involved in the heat response of wheat (Xin et al., 2010; Khraiwesh et al., 2012). Also, the expression levels of 20 known miRNAs presented a significant difference between heat-tolerant and heat-sensitive broccoli, by sRNA sequencing (Chen et al., 2015).

*Arabidopsis* miR156 regulates adaptation to recurring heat stress (heat shock memory) through SQUAMOSA promoter binding protein-like (*SPL*) genes (Stief et al., 2014). The miR39-copper/zinc superoxide dismutase (*CSD*) pathway can regulate the contents of radical oxygen species (ROS) in *Arabidopsis*, thereby mediating *HSF* expression (Guan et al., 2013). Transgenic *Arabidopsis* plants overexpressing miR400 (Yan et al., 2012) and miR173/*TAS1* (Li et al., 2014) were both more sensitive to heat stress than wild-type (WT) plants. In sunflower (*Helianthus annuus*), *HaWRKY6* and miR396 presented opposing expression profiles at high temperature, and the miRNA-resistant versions of *HaWRKY6* showed more tolerance to heat stress than the WT (Giacomelli et al., 2012).

miR160 and its target genes (*ARFs*) play vital roles in plant growth and development. Repression of *ARF10* and *ARF16* by miR160 has been shown to mediate seed germination in *Arabidopsis* (Liu et al., 2007, 2013). Meanwhile, the hypocotyl growth of *Arabidopsis* was negatively regulated by *ARF17* (Mallory et al., 2005). In tomato (*Solanum lycopersicum*), expression of a short tandem target mimic of miR160 RNA (STTM160) (Damodharan et al., 2016) and miR160-insensitive *SlARF10* (Hendelman et al., 2012) affected floral organ development. miR160 might also participate in plant responses to environmental stress. For instance, repression of *ARF10* by miR160 is involved in regulating leaf water balance in tomato (Liu et al., 2016). Moreover, it has been suggested that miR160 could have a role in the response of plants to fungal disease (Pinweha et al., 2015) and virus infection (Navarro et al., 2008; Khraiwesh et al., 2012; Wang and Luan, 2015).

Next-generation sequencing and northern blot analyses have revealed that the regulation of miR160 expression in response to heat occurs in various plants besides *Arabidopsis* (Zhong S. H. et al., 2013), wheat (Kumar et al., 2015), and barley (Kruszka et al., 2014). Under heat stress, both, miR160- and miR157-mediated auxin signaling regulate male sterility in cotton (*Gossypium hirsutum*) (Ding et al., 2017). However, the role of miR160 in heat regulation is still not fully elucidated. In this study, miR160 and its targets were investigated in plants under heat stress. Transgenic *Arabidopsis* expressing additional *miR160 precursor a* (*pre-miR160a*) and artificial miR160 target mimicry (*mimic160*) were constructed to advance our understanding of the functions of miR160 in plants in response to heat. Plant development and the gene expression of *HSPs* in the transgenic plants were altered to regulate thermotolerance of the plant in response to adverse heat conditions.

## Materials and methods

### Plant materials

*Arabidopsis thaliana* (Col-0) was grown in a growth chamber at 22°C under 16 h light/8 h dark with cool fluorescent light at 100 μmolm^−2^ s^−1^. Transgenic plants overexpressing *pre-miR160a* (160OE) and expressing miR160 target mimicry (MIM160) were created by the floral dip method (Clough and Bent, 1998). T-DNA insertion lines of *ARF10* (AT2G28350), *ARF16* (AT4G30080), *ARF17* (AT1G77850), and *HSP101* (AT1G74310) are CS24611 (*arf10-1*), SALK_021432 (*arf16-3*), SALK_138426 (*arf17-2*), and CS16284 (*hot1-3*), respectively, and they were obtained from the Arabidopsis Biological Resource Center (ABRC, Ohio State University). All seeds used in the same experiments of this study are from plants growing at the same time under the same conditions and stored identically.

### Plasmid construction

The fragment of *miR160 precursor a* (*pre-miR160a*) was obtained by PCR with Arabidopsis cDNAs as templates and BamHI-premiR160 F/SacI-premiR160 R (Supplemental Table S1) as primer sets. The sequence of miR160 target mimic inhibitor (*mimic160*) was modified from the sequence of *IPS1* gene (Franco-Zorrilla et al., 2007). The fragment of *mimic160* was amplified by PCR with a synthetic single-strand DNA containing *mimic160* sequence (MDBio, Inc.; Supplemental Table S1) as a template and BamHI-mimic F/SacI-mimic R (Supplemental Table S1) as primer sets. PCR amplified *pre-miR160a* and *mimic160* fragments were inserted into the yT&A vector (Yeastern Biotech). Then, they were cleaved by *Bam*HI and *Sac*I, and inserted into the region between the 35S promoter and the terminator in pBI221 vector to create *35S-pre-miR160a-terminator* and *35S-mimic160-terminator*. They were further cloned into binary vector pCAMBIA1300 and pCAMBIA2300, respectively. Then, pCAMBIA1300-*pre-miR160* and pCAMBIA2300-*mimic160* were transformed into *Agrobacterium tumefaciens* strain LBA4404 to performed floral dip transformation.

### Gene expression analyses

Total RNA of the seedling was isolated by using Trizol reagent (Invitrogen) according to the manufacturer's instructions. DNase I (Ambion) treated-RNAs were reverse transcribed with the reaction of MMLV reverse transcriptase (Invitrogen) with T_25_VN primer (Supplemental Table S1) at 37°C for 90 min to produce cDNA. Further, the semi-quantitative or quantitative PCR for expression analyses were used to amply cDNA to detect expression of miR160 precursor genes, *pre-miR160a* (AT2G39175), *pre-miR160b* (AT4G17788), and *pre-miR160c* (AT5G46845), miR160 target genes, *ARF10* (AT2G28350), *ARF16* (AT4G30080), and *ARF17* (AT1G77850), and RNA sequencing-selected genes, *HSP101* (AT1G74310), *HSP70B* (AT1G16030), *HSP21* (AT4G27670), *HSP17.6A* (AT5G12030), and *HSP17.6II* (AT5G12020). Primer sets pre-miR160a F/pre-miR160a R, pre-miR160b F/pre-miR160b R, pre-miR160c F/pre-miR160c R, ARF10 F/ARF10 R, ARF16 F/ARF16 R, ARF17 F/ARF17 R, HSP101 F/HSP101 R, HSP70B F/HSP70B R, HSP21 F/HSP21 R, HSP17.6A F/HSP17.6A R, HSP17.6II F/HSP17.6II R, and Actin F/Actin R (Supplemental Table S1) were used to analyze the expression levels of *pre-miR160a, pre-miR160b, pre-miR160c, ARF10, ARF16, ARF17, HSP101, HSP70B, HSP21, HSP17.6A, HSP17.6II*, and *Actin* (AT3G18780), respectively. In quantitative PCR assays, the amplification reactions contain 1 × SYBR Green Supermix (Bio-Rad), 125 nM primers, and 100 ng cDNA. Data are normalized by the expression levels of *Actin* gene, and are shown as the relative expression levels for at least three biological assays, which include at least three technical replicates.

### miRNA analysis

miRNA blot assays were performed based on the procedure described by Lin et al. (2012, 2013). Total RNAs (20 μg) were analyzed on a 12% polyacrylamide gel with 8 M urea. After the separation of RNA, gel was blotted to a Hybond-NX membrane (GE Healthcare), and the membrane was UV cross-linked (Pall et al., 2007). The blot was hybridized by the radio-labeled antisense miR160 probes synthesized by *in vitro* transcription. T3 RNA polymerase (Promega) with T3-miR160 (Supplemental Table S1), which annealed to T3 top strand (Supplemental Table S1), was used for *in vitro* transcription. After hybridization, the blot was washed twice in wash buffer 1 [2 × SSC and 0.1% (w/v) SDS] at 55°C for 15 min and once in wash buffer 2 (0.2 × SSC and 0.1% (w/v) SDS) at 55°C for 15 min. The radioactive signal was subsequently displayed on the Typhoon 9400 (GE Healthcare). In addition, the stripped blots were re-hybridized by the radio-labeled 5S rRNA probe. PCR with primer sets 5S rRNA F/5S rRNA R (Supplemental Table S1) and cDNA of sweet potato was used to produce this probe. The presented 5S rRNA signals acted as internal controls for miRNA bolt assays.

### Seed germination assay

Arabidopsis seeds were incubated in 1% bleach with 0.1% triton X-100 for 10 min for surface sterilization. Sterilized seeds were germinated in the half-strength Murashige and Skoog (MS) agar medium containing 1% sucrose and incubated at 4°C for 3 days. After imbibition, the plates were incubated at 22°C for 2 h. Then seeds were treated at 50°C for 2 h. After 4 days recovery at 22°C, the seed germinations were recorded. The treatment of heat stress was performed in a water bath instrument.

### Survival percentage analysis

After surface sterilized Arabidopsis seeds were imbibed at 4°C for 3 days, they were incubated in the half-strength MS agar medium containing 1% sucrose at 22°C for 7 days. Then, the 7-day-old seedlings were treated with heat stress at 44°C for 24 or 30 min. After 14 days recovery at 22°C, photographs were taken and the survival percentages were recorded. The *hot1-3* plant, a heat-sensitive mutant, was used as a negative control. Water bath was used for heat treatment.

### Hypocotyl elongation measurement

Arabidopsis seeds sterilized by 1% bleach with 0.1% triton X-100 were imbibed at 4°C for 3 days. Then, they were incubated in the half-strength MS agar medium containing 1% sucrose in the dark at 22°C for 3 days. The hypocotyl lengths of 3-day-old seedlings were measured first. Seedlings were then incubated at 37°C for 1.5 h, recovered at 22°C for 2 h, and then treated with heat stress at 45°C for 3 h. The hypocotyl lengths were recorded 2 days later. The differences of hypocotyl lengths before and after heat treatment were calculated. The *hot1-3* mutant was used as a negative control. The treatment of heat stress was performed in a water bath.

### Rachis length experiment

The 21-, 24-, or 28-day-old plants were incubated in a plant growth chamber at 30°C for 24, 21, or 17 days, respectively. Then, photographs were taken and the rachis lengths were recorded. The plants grown in normal condition were also analyzed for comparison.

### RNA sequencing

The RNA sequencing was performed by Welgene Biotech. (http://www.welgene.com.tw). RNA library preparation and sequencing were executed according to the manufacture's protocol from Illumina. Agilent's SureSelect Strand Specific RNA Library Preparation Kit was used for library construction. Then, the TruSeq SBS Kit was used for sequencing by the Solexa platform. After sequencing, low-quality data were filtered. Then, qualified reads were analyzed using TopHat/Cufflinks for the estimation of gene expression (Trapnell et al., 2012). Genes differentially expressed between WT and transgenic plants were further analyzed by RT-qPCR. *HSP101, HSP70B, HSP21, HSP17.6A*, and *HSP17.6II* were chosen for further investigation. The raw data of RNA sequencing were uploaded to Gene Expression Omnibus (GEO, http://www.ncbi.nlm.nih.gov/geo). The accession number is GSE103041.

## Results

### Regulation of miR160 and its targets under heat stress

**Figure 1:**
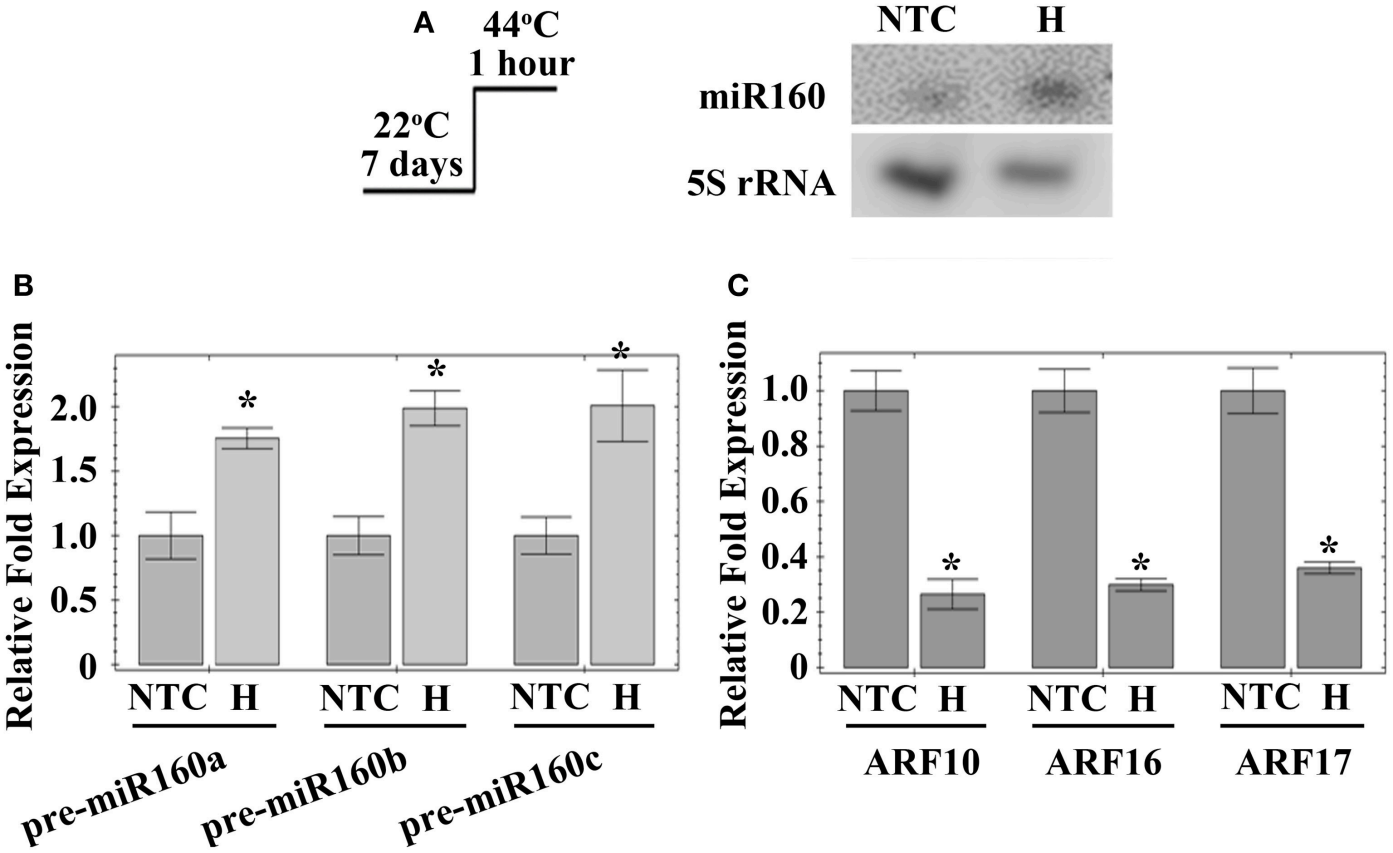
Expression of miR160, miR160 precursor genes and miR160 targets under heat stress. The 7-day-old Arabidopsis was treated with heat stress (H) at 44°C for 1 h. Seedlings without heat stress were included for comparison (NTC). The total RNAs from these seedlings were extracted and analyzed. **(A)** Expression of miR160 and 5S rRNA was detected by northern blottings, and 5S rRNA levels were used as internal controls. **(B)** Expression of miR160 precursor genes, *pre-miR160a, pre-miR160b*, and *pre-miR160c*, was analyzed by quantitative RT-PCR. These data were normalized to *Actin* gene expression and their ratios relative to those of plants without heat stress are shown as the relative expression levels. **(C)** Expression of *ARF10, ARF16*, and *ARF17*, the miR160 targets, was analyzed using quantitative RT-PCR. These data were normalized to *Actin* gene expression and their ratios relative to those of plants without heat stress are shown as the relative expression levels. Statistic differences between plants treated with and without heat stress are marked with *star* according to hypothesis test (^*^P < 0.05). Treatment conditions are shown on **(A)**. Data are presented as mean ± standard deviation (*n* = 3).

As mentioned above, heat affects miR160 expression in various plants (Zhong S. H. et al., 2013; Kruszka et al., 2014; Kumar et al., 2015; Ding et al., 2017). In *Arabidopsis*, deep sequencing of sRNAs determined that miR160 expression is stimulated at 30°C (Zhong S. H. et al., 2013). Microarray analysis demonstrated heat represses the expression of *ARF16*, one of the miR160 targets, in *Arabidopsis* (Li et al., 2014). However, the function of miR160 in the heat responses of *Arabidopsis* remains unknown. Hence, the current study investigated the regulation of miR160 in *Arabidopsis* under heat stress, using northern blotting. When *Arabidopsis* seedlings were treated at 44°C for 1 h, mature miR160 was induced (Figure 1A). Mature miR160 is cleaved from precursor miRNAs, pre-miR160a, pre-miR160b, and pre-miR160c (Reinhart et al., 2002). Quantitative RT-PCR identified that the expression of all three miR160 precursor genes was increased by heat stress (Figure 1B), indicating that induction of miR160 was generated from its precursors. In *Arabidopsis, ARF10, ARF16*, and *ARF17* are miR160 target genes (Rhoades et al., 2002). Consequently, the expression of miR160 targets was also analyzed under the same condition, establishing that *ARF10, ARF16*, and *ARF17* expressions were significantly repressed after heat treatment (Figure 1C). These results indicated that heat stress induced the generation of mature miR160 from its precursors, to suppress the expression of *ARF10, ARF16*, and *ARF17*.

### Expression of miR160 target genes in 160OE and MIMI160 transgenic plants

**Figure 2:**
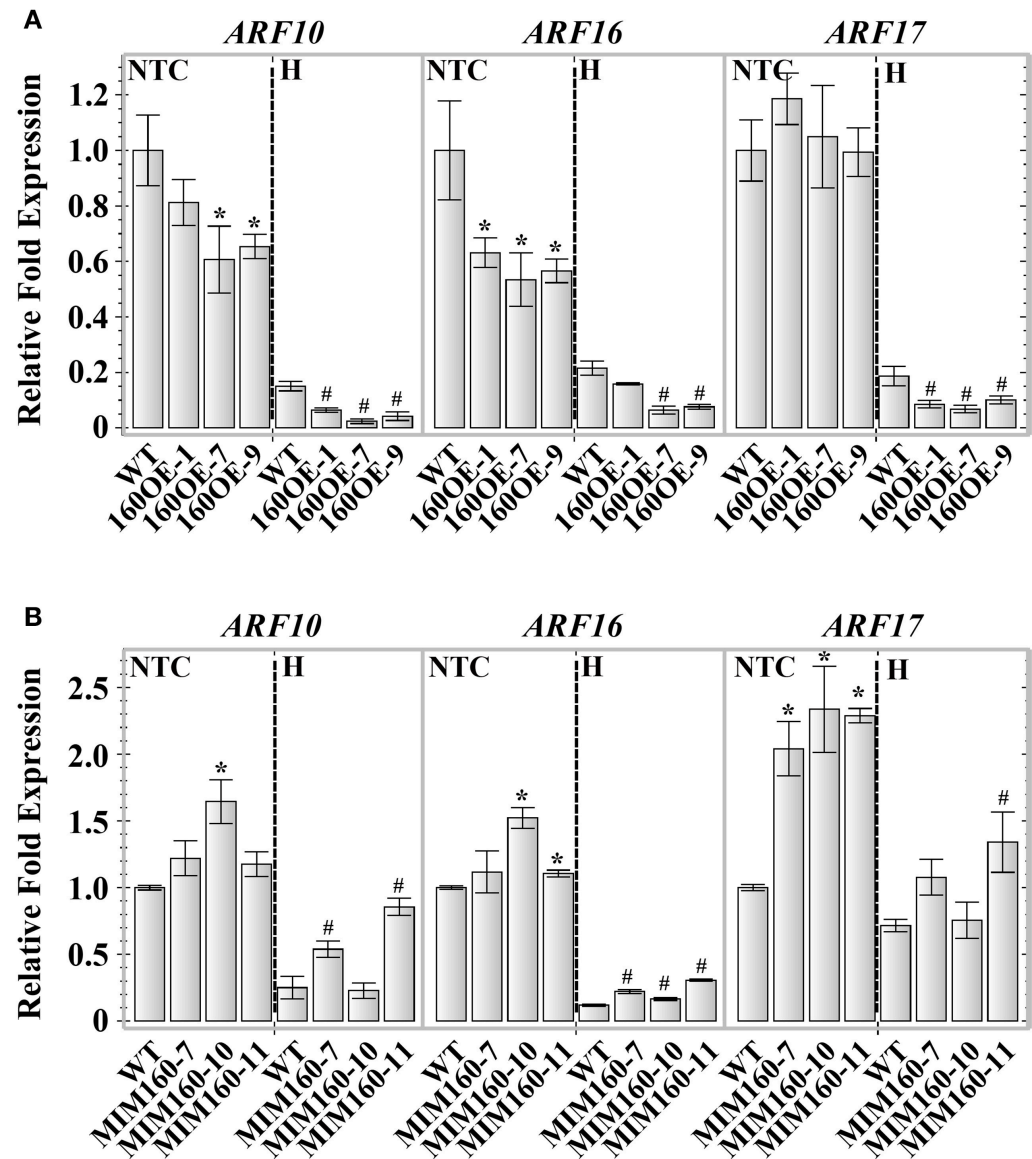
Expression of *ARF10, ARF16*, and *ARF17* in WT, 160OE, and MIM160 plants under heat stress. The 7-day-old 160OE and MIM160 seedlings were treated with heat stress (H) at 44°C for 1 h and 25 min, respectively. Seedlings without heat stress were included for comparison (NTC). The total RNAs from these seedlings were extracted and analyzed. The expression of miR160 targets, *ARF10, ARF16*, and *ARF17*, in 106OE **(A)** and MIM160 **(B)** was analyzed using quantitative RT-PCR, and normalized to the levels of *Actin* expression. Their ratios relative to those of WT are shown as the relative expression levels. Statistic differences between WT and transgenic plants are marked with *star* according to hypothesis test (^*,#^*P* < 0.05; ^*^plants were grown in control condition; ^#^plants were treated with heat stress). Data are presented as mean ± standard deviation (*n* = 3).

Transgenic plants overexpressing *miR160 precursor a* (160OE) and artificial miR160 target mimicry (MIM160) were produced to study the roles of miR160 under heat stress in this study. The expression of *miR160 precursor a* and mature miR160 was significantly increased in 160OE compared to the WT plant (Supplemental Figures S1A,B). Without heat treatment, the expression of miR160 targets, *ARF10* and *ARF16*, was repressed in 160OE plants (Figure 2A). Under heat stress, the expression of miR160 targets, *ARF10, ARF16*, and *ARF17*, in 160OE was lower relative to the WT (Figure 2A). In order to inhibit miR160 function, the artificial miR160 target mimic inhibitor (*mimic160*) modified from *induced by phosphate starvation-1* (*IPS1*) gene was used (Franco-Zorrilla et al., 2007), and the expression of *mimic160* was only detected in MIM160 plants rather than the WT plant (Supplemental Figure S2). In MIM160 plants expressing *mimic160*, the expression of *miR160 precursor a* was slightly altered, and mature miR160 was suppressed (Supplemental Figures S3A,B). According to the characteristics of the target mimic inhibitor, artificial mimic160 might elevate the expression of miR160 targets. Without heat treatment, expression of *ARF10, ARF16*, and *ARF17* was increased in MIM160 compared to WT (Figure 2B). In contrast, under heat stress, the repression levels of *ARF10, ARF16*, and *ARF17* were decreased in MIM160 in comparison to WT (Figure 2B), indicating the function of miR160 was reduced in the presence of *mimic160*.

### Seed germination, survival percentages, hypocotyl elongation lengths, and rachis lengths of 160OE and MIMI160 plants

**Figure 3:**
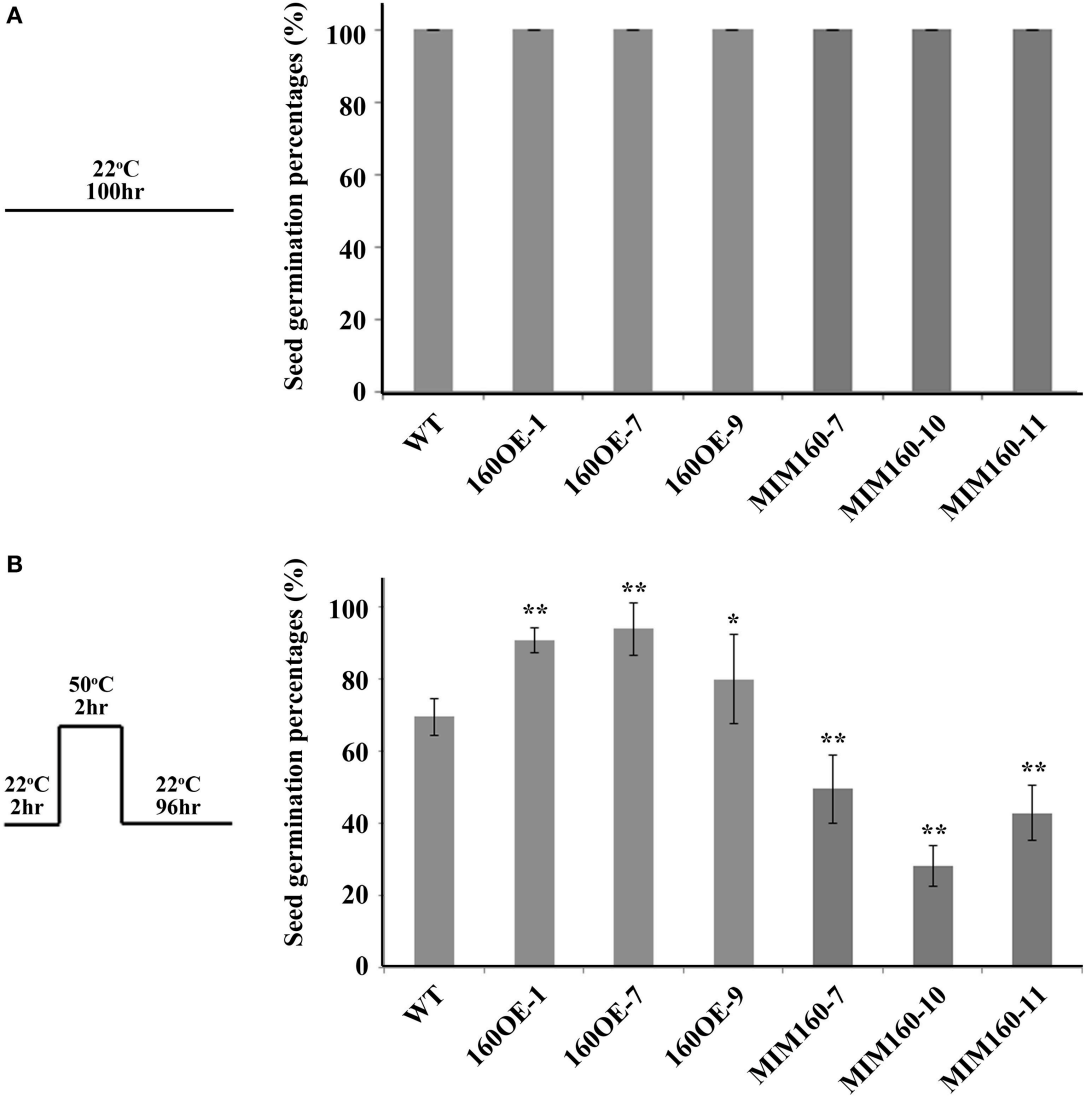
Seed germination percentages of WT, 160OE, and MIM160 plants under heat stress. Seed germination percentages of WT, 160OE, and MIM160 grown under normal condition at 22°C for 100 h were presented in **(A)**, and these seeds treated with heat stress were analyzed and presented in **(B)**. After imbibition, these seeds of WT, 160OE, and MIM160 were incubated at 22°C for 2 h. Then, they were treated at 50°C for 2 h. After 22°C recovery for 4 days, seed germination percentages were recorded. The treatment conditions are shown on the top. Statistic differences between WT and transgenic plants are marked with *star* according to Student's test (^*^P < 0.05; ^**^P < 0.01). The error bars are indicated as the standard deviation for at least five biological assays (*n* ≥ 5).

Seed germination percentages of WT, 160OE, and MIM160 were further measured after heat treatment. At 22°C, seeds of all plants were normally germinated (Figure 3A). However, about 73% of the seeds of WT were germinated when they were treated at 50°C for 2 h and then recovered at 22°C for 4 days (Figure 3B). Under the same condition, about 80–94% of the seeds of 160OE were germinated (Figure 3B), indicating the increase of miR160 expression could elevate seed germination percentages after heat treatment. Interestingly, when seeds of MIM160 were treated with heat, only about 28–49% seed germination occurred (Figure 3B). Thus, inhibition of miR160 function could significantly decrease seed germination percentages under heat stress. These findings suggested that miR160 repressed its target genes to elevate the heat tolerance of *Arabidopsis* during seed germination.

**Figure 4:**
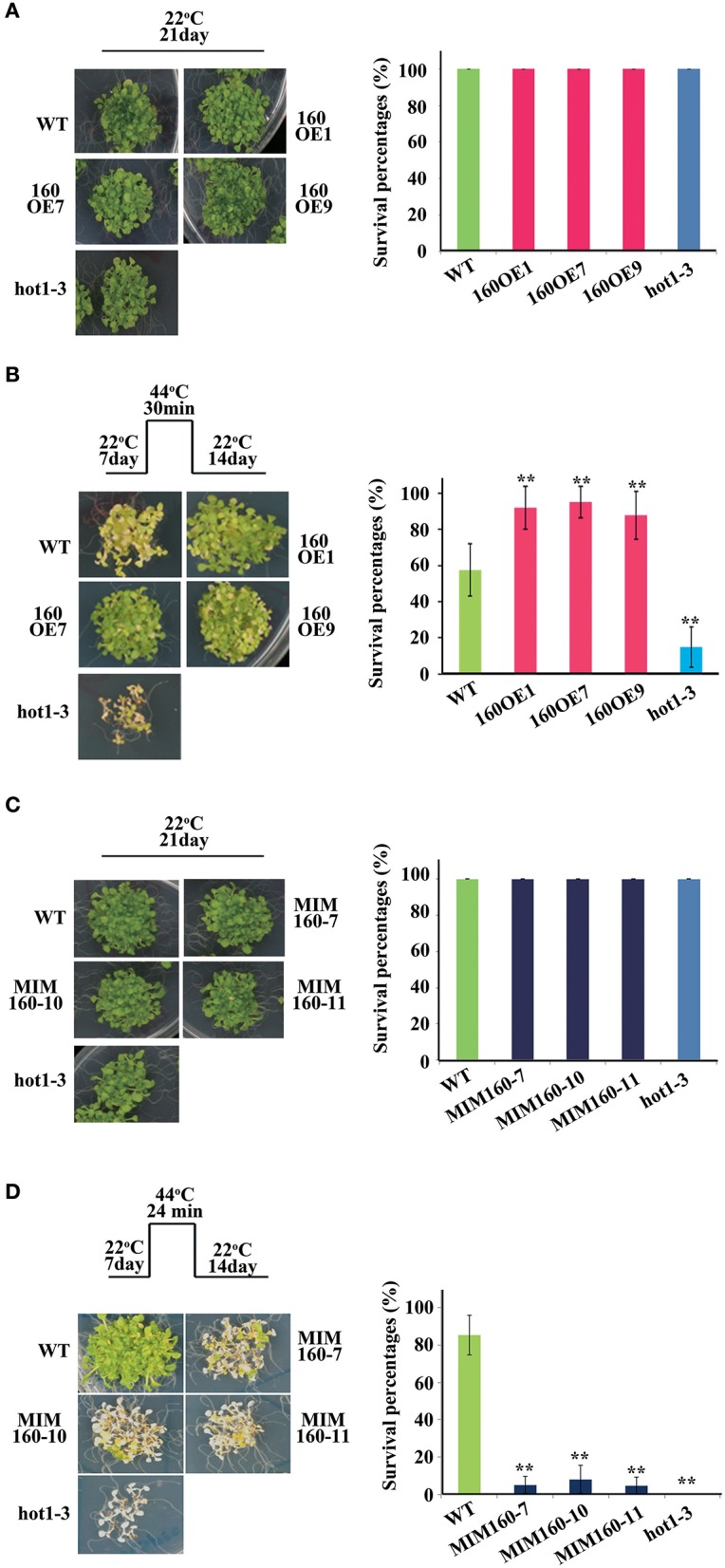
Survival percentages of WT, 160OE, and MIM160 under heat stress. Survival percentages of 160OE **(A,B)** and MIM160 **(B,C)** seedlings treated with and without heat stress were evaluated. The survival percentages of 7-day-old 160OE **(A)** and MIM160 **(C)** seedlings incubated at 22°C for 14 days were evaluated. In addition, the 7-day-old 160OE **(B)** and MIM160 **(C)** seedlings were treated with heat stress at 44°C for 30 and 24 min, respectively. After 22°C recovery for 14 days, survival percentages were recorded. Treatment conditions and photographs of plants are shown on the left, and the quantitative data are shown on the right. Statistic differences between WT and transgenic plants are marked with *star* according to Student's test (^**^P < 0.01). The error bars are indicated as the standard deviation for at least five biological assays (*n* ≥ 5). The *hot1-3* mutant acts as a negative control.

Survival percentages upon heat stress were also analyzed (Figures 4A–D). At 22°C for 21 days, survival percentages of all WT, 160OE, and MIM160 seedlings were nearly 100% (Figures 4A,C). The 7-day-old WT and 160OE seedlings were heat-stressed by exposure to 44°C for 30 min and recovered at 22°C for 14 days. About 60% WT and 90% 160OE plants were alive (Figure 4B). However, the survival percentage of MIM160 plants was substantially reduced to about 10% after they were treated under the same condition, except the 44°C treatment was decreased to 24 min (Figure 4D). It might indicate that the expression of miR160 is needed for the plant to survive when in heat stress.

**Figure 5:**
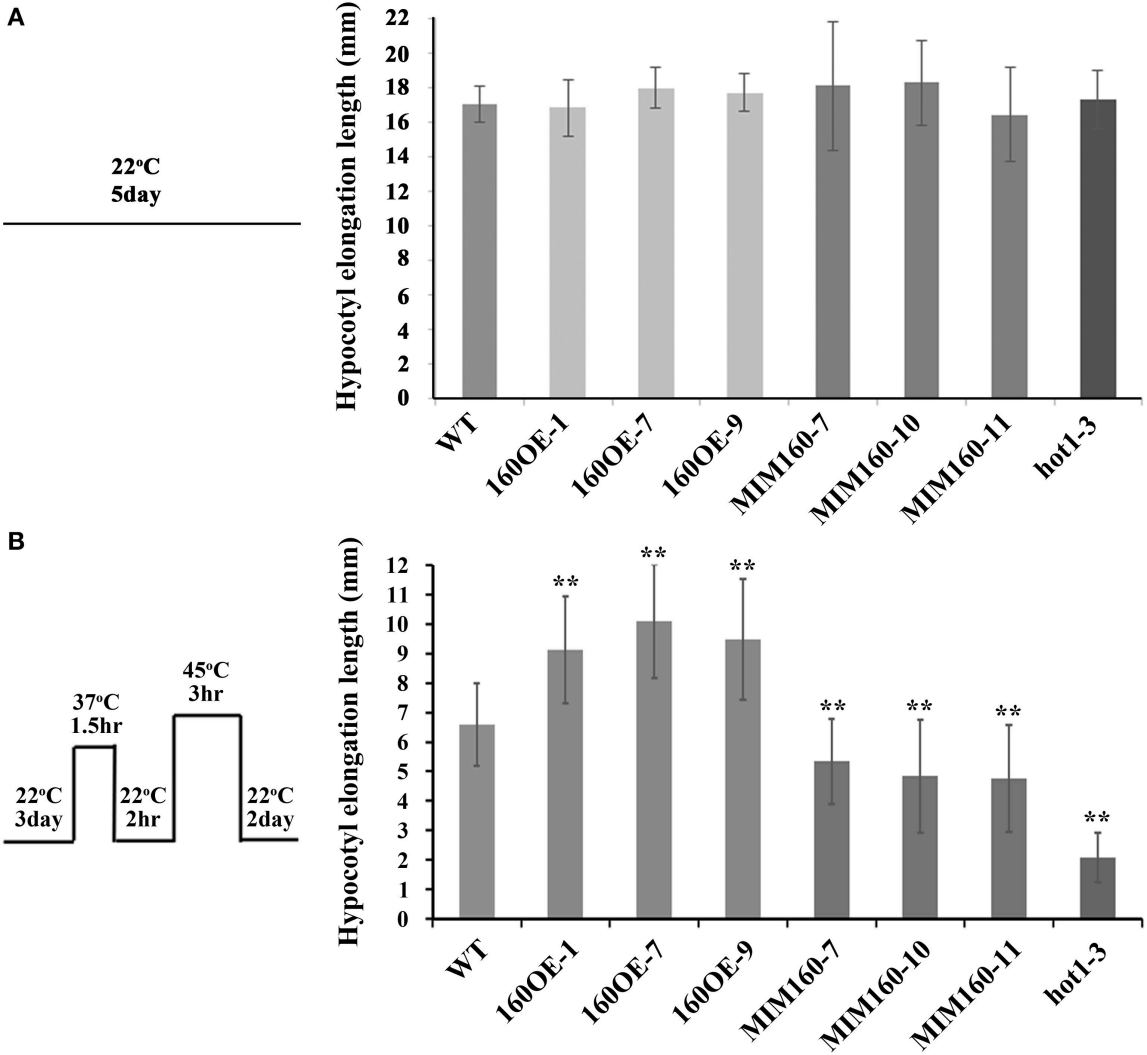
Hypocotyl elongation lengths of WT, 160OE, and MIM160 under heat stress. The hypocotyl elongation lengths of WT, 160OE, and MIM160 seedlings grown at 22°C in darkness were presented in **(A)**, and those under heat stress were measured and presented in **(B)**. The 3-day-old WT, 160OE, and MIM160 seedlings grown in darkness were incubated at 37°C for 1.5 h. After 22°C recovery for 2 h, these seedlings were treated with heat stress at 45°C for 3 h. Then, hypocotyl lenghts were recorded 2 days later. The hypocotyl lengths of 3-day-old seedlings were also measured. The differences of hypocotyl lengths before and after heat treatment were calculated. Treatment conditions are shown on the top. Statistic differences between WT and transgenic plants are marked with *star* according to Student's test (^**^P < 0.01). The error bars are indicated as the standard deviation for at least twenty biological assays (*n* ≥ 20). The *hot1-3* mutant acts as a negative control.

The hypocotyl elongation lengths were also recorded. At 22°C, the hypocotyl elongation lengths among WT, 160OE, and MIM160 seedlings showed no significant difference (Figure 5A). The 3-day-old seedlings were pretreated at 37°C for 1.5 h, recovered at 22°C for 2 h, and then treated with heat stress at 45°C for 3 h. The hypocotyl elongation length was measured 2 days later. The hypocotyl elongation lengths of 160OE and MIM160 plants were significantly longer and shorter than those of WT (Figure 5B), respectively, indicating miR160 was involved in the growth of hypocotyl during heat stress.

**Figure 6:**
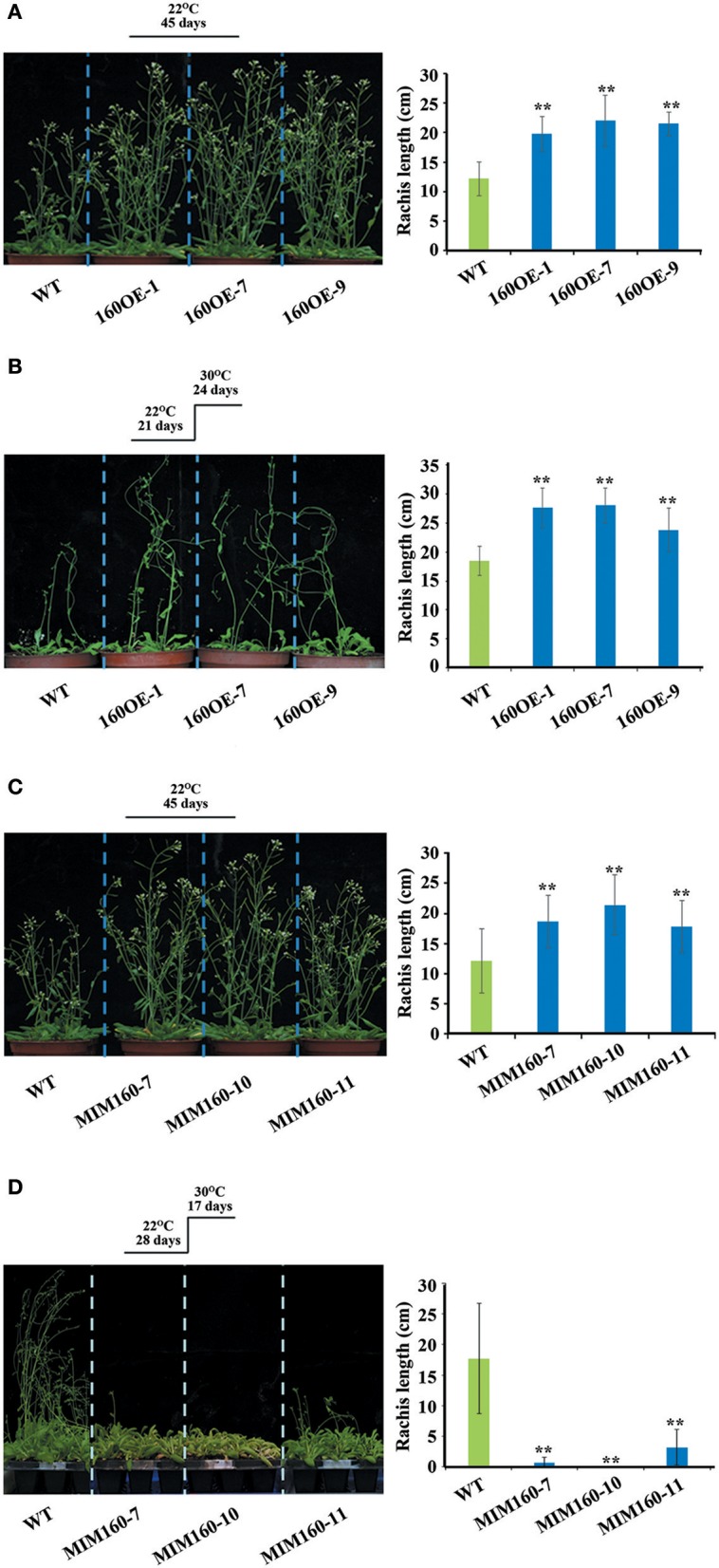
Rachis lengths of WT, 160OE, and MIM160 under heat stress. The 21-day-old WT and 160OE plants were incubated at 22°C **(A)** and 30°C **(B)** for 24 days. In addition, the 28-day-old WT and MIM160 plants were also incubated at 22°C **(C)** and 30°C **(D)** for 17 days. Then, their rachis lengths were measured. Treatment conditions and photographs of plants are shown on the left; quantitative data are shown on the right. Statistic differences between WT and transgenic plants are marked with *star* according to Student's test (^**^P < 0.01). The error bars are indicated as the standard deviation for at least six biological assays (*n* ≥ 6).

In addition, the rachis lengths of WT, 160OE, and MIM160 were analyzed. The rachis lengths were longer in 160OE than WT, when the plants were grown at 22 or 30°C (Figures 6A,B). The rachis lengths of MIM160 grown at 22°C were also longer than those of WT (Figure 6C). However, rachis lengths of MIM160 at 30°C were much shorter relative to WT (Figure 6D), indicating the expression of miR160 might profoundly affect rachis development. According to the seed germination percentages, survival percentages, hypocotyl elongation lengths, and rachis lengths, miR160 played important roles in the adaptation of *Arabidopsis* to heat stress.

### Effects of *ARF10, ARF16*, and *ARF17* on seed germination, survival percentages, hypocotyl elongation lengths, and rachis lengths

**Figure 7:**
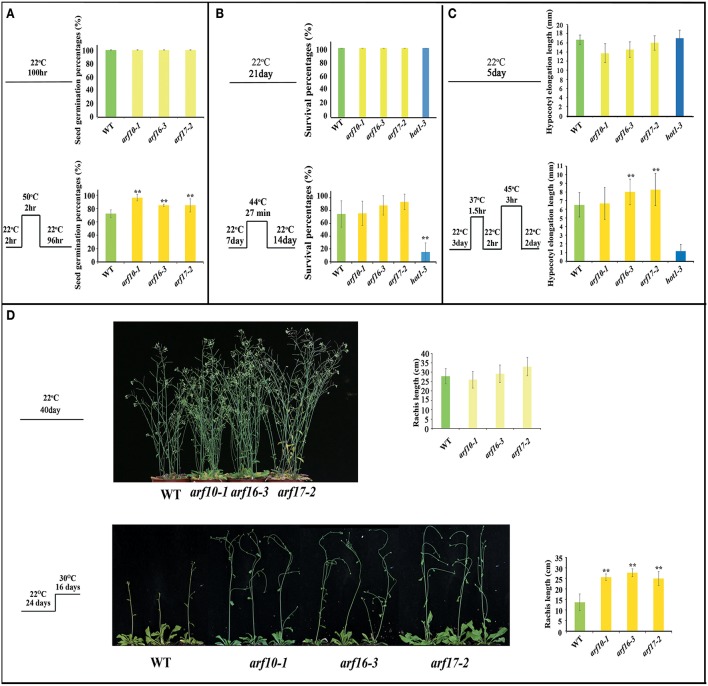
Seed germination percentages, survival percentages, hypocotyl elongation lengths, and rachis lengths of WT and the T-DNA insertion mutants *arf10-1, arf16-3*, and *arf17-2* under heat stress. WT and the T-DNA insertions *arf10-1, arf16-3*, and *arf17-2* were used to examine thermotolerance abilities. The conditions of heat treatments for seed germination percentages **(A)**, survival percentages **(B)**, hypocotyl elongation lengths **(C)**, and rachis lengths **(D)** are similar to those mentioned in Figures 3–6, respectively. Treatment conditions are shown on the left. Statistic differences between WT and transgenic plants are marked with *star* according to Student's test (^**^P < 0.01). The error bars are indicated as the standard deviation for at least eight biological assays (*n* ≥ 8). The *hot1-3* mutant acts as a negative control.

160OE and MIM160 plants revealed better and worse thermotolerance than the WT plant, respectively (Figures 4–6). In *Arabidopsis, ARF10, ARF16*, and *ARF17* are miR160 targets (Rhoades et al., 2002). Hence, *arf10* (CS24611: *arf10-1*), *arf16* (SALK_021432: *arf16-3*), and *arf17* (SALK_138426: *arf17-2*) T-DNA mutants from ABRC (Supplemental Figure S4) were used to examine their tolerances to heat stress. Gene expression of *ARF10, ARF16*, and *ARF17* was largely repressed in *arf10, arf16*, and *arf17* mutants, respectively (Supplemental Figures S4B,D). Their seed germination percentages, survival percentages, hypocotyl elongation lengths, and rachis lengths in the heat stressed plants were analyzed (Figures 7A–D). There were no significant differences in these phenotypes among WT, *arf10, arf16*, and *arf17* mutants grown under the control condition (Figures 7A–D). Nonetheless, after heat treatment, the seed germination percentages of WT, *arf10-1, arf16-3*, and *arf17-2* plants were about 71.2, 95.8, 85.5, and 84.8%, respectively (Figure 7A). Thus, the reduction of *ARF10, ARF16*, and *ARF17* expression could elevate seed germination during the heat response. WT, *arf10-1, arf16-3*, and *arf17-2* plants presented no significant differences in their survival percentages (Figure 7B). The hypocotyl elongation lengths were longer in *arf16-3* and *arf17-2* plants than the WT (Figure 7C), indicating loss of function of *ARF16* and *ARF17* could enhance the hypocotyl elongation of plants during the stress response. Under heat stress, the *arf10-1, arf16-3*, and *arf17-2* plants displayed higher rachis lengths than the WT (Figure 7D). Therefore, the phenotypes of *arf10, arf16*, and *arf17* mutants were similar to those of 160OE plants in heat stress, and ARF10, ARF16, and ARF17 negatively regulated the heat tolerance of *Arabidopsis*.

### Expression of heat shock protein genes in transgenic plants

**Figure 8:**
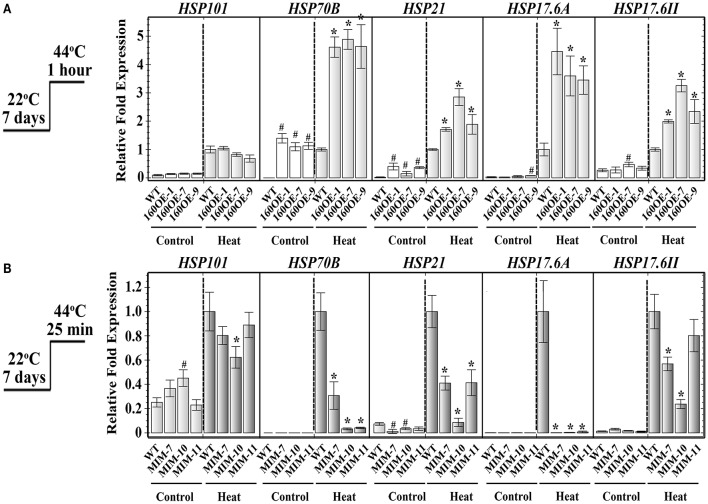
Expression of *HSP*s in WT, 160OE, and MIM160 plants under heat stress. The 7-day-old 160OE **(A)** and MIM160 **(B)** seedlings were treated with heat stress at 44°C for 1 h and 25 min, respectively. Seedlings without heat stress were included for comparison. The total RNAs from these seedlings were extracted and analyzed by RT-PCR. The expression of *HSP101, HSP21, HSP18, HSP17.6A*, and *HSP17.6II* was analyzed using quantitative RT-PCR, and normalized to the levels of *Actin* expression. Their ratios relative to those of heat-treated WT are shown as the relative expression levels. Statistic differences between WT and transgenic plants are marked with *star* according to hypothesis test (^#,*^P < 0.05; ^#^plants were grown in control condition; ^*^plants were treated with heat stress). Treatment conditions are shown on the left. Data are presented as mean ± standard deviation (*n* = 3).

RNA sequencing was used to analyze the transcriptomes of WT and 160OE plants with/without heat stress, to decipher the miR160-ARF mechanism in the responses to heat stress. After RNA sequencing and filtering low-quality data, TopHat and Cufflinks were used for gene expression estimation (Trapnell et al., 2012). It was found that FPKM (fragments per kilobase of transcript per million mapped reads; Table 1a) and relative expression ratios (Table 1b) of several HSPs and ABA-related genes were much higher in the 160OE than WT plant under the normal condition and heat treatment. HSPs are regarded as the central components of the heat stress response in plants (Nover and Scharf, 1997; Kotak et al., 2007). Several transcriptomes-selected *HSP* genes, including *HSP101, HSP70B, HSP21*, and *HSP17.6A*, and *HSP17.6II*, were further analyzed by qRT-PCR. The expression of *HSP70B, HSP21, HSP17.6A*, and *HSP17.6II* was increased in 160OE plants relative to WT (Figure 8A), indicating qRT-PCR results were similar to those acquired from RNA sequencing. Additionally, these genes expression levels in MIM160 plants were analyzed by qRT-PCR, and the expression of *HSP70B, HSP21, HSP17.6A*, and *HSP17.6II* in MIM160 plants was decreased after heat treatment (Figure 8B). These results implied that miR160 might mediate *HSP* expression to regulate the heat stress responses in *Arabidopsis*. To advance the understanding of *ARF10, ARF16*, and *ARF17* in heat stress responses, transcriptomes of *arf10-1, arf16-3*, and *arf17-2* T-DNA insertion plants under heat stress were also dissected. RNA sequencing results revealed FPKM (Table 1a) and relative expression ratios (Table 1b) of *HSP101, HSP70B, HSP21*, and *HSP17.6A*, and *HSP17.6II* in *arf10-1, arf16-3*, or *arf17-2* were enhanced after heat treatment compared to those in WT. qRT-PCR data also demonstrated that the expression levels of these genes were increased in *arf10-1, arf16-3*, or *arf17-2* relative to WT (Supplemental Figure S5). Thus, collectively, these results indicated the expression levels of miR160 targets, namely *ARF10, ARF16*, and *ARF17*, altered *HSP* expression and further regulated the heat stress responses in *Arabidopsis*.

**Table 1:** Expression data **(a)** and Relative expression ratio **(b)** of genes related to HSP and ABA analyzed by RNA sequencing.

	**Gene_id**	**Gene name**	**WTcon**	**160OEcon**	***arf10*con**	***arf16*con**	***arf17*con**	**WTH**	**160OEH**	***arf10*H**	***arf16*H**	***arf17*H**
**(a)**												
HSP	AT1G74310	HSP101	1.97077	2.28089	0.986023	2.00806	2.23759	9.5854	43.4656	12.8303	27.0586	19.4094
	AT5G52640	HSP90-1	1.74728	5.6307	1.50067	3.53854	7.56977	25.0851	110.021	44.4897	69.1907	59.0706
	AT1G16030	HSP70B	1.10214	0.854592	0.75951	0.909039	1.44125	1.62456	10.8445	2.00188	5.69133	3.96703
	AT1G52560	HSP26.5	0.05635	0.194549	0.453153	0.0001	0.459592	0.176774	1.23449	0.343245	0.838633	0.512882
	AT4G10250	HSP22.0	0.0001	0.0639221	0.0001	0.0001	0.0422322	1.21973	2.97422	1.4572	3.10819	2.72171
	AT4G27670	HSP21	0.18225	0.136354	0.158792	0.574124	0.450435	0.278769	0.576763	0.370047	1.35617	0.760279
	AT5G12030	HSP17.6A	0.55815	1.48772	0.638301	1.12094	1.96582	30.3088	55.4766	32.6397	64.0327	53.4223
	AT5G12020	HSP17.6II	4.61155	8.15528	7.76221	5.1507	8.39292	28.0021	58.8752	47.0737	68.1999	47.6183
	AT2G29500	HSP17.6B	1.44448	6.23506	2.0331	2.57278	5.06686	14.7018	23.8681	21.1852	33.9697	23.4507
ABA- relate genes	AT1G70800	CAR6	0.0001	0.246802	0.215569	0.0001	0.163066	0.420489	0.260999	0.200945	0.136379	0.125114
	AT1G70910	DEP	0.0001	0.256557	0.0001	0.108024	0.0847514	0.349678	0.271302	0.0001	0.283523	0.130046
	AT2G40220	ABI4	0.0001	0.0936679	0.218163	0.0394391	0.0309424	0.063833	0.396204	0.0001	0.051756	0.0001
	AT5G52310	RD29A	0.25066	0.887218	0.252004	0.710685	1.04367	1.03228	3.47823	1.08057	2.12833	1.20656
**(b)**												
HSP	AT1G74310	HSP101	1.00	1.16	0.50	1.02	1.14	4.86	22.06	6.51	13.73	9.85
	AT5G52640	HSP90-1	1.00	3.22	0.86	2.03	4.33	14.36	62.97	25.46	39.60	33.81
	AT1G16030	HSP70B	1.00	0.78	0.69	0.82	1.31	1.47	9.84	1.82	5.16	3.60
	AT1G52560	HSP26.5	1.00	3.45	8.04	0.00	8.16	3.14	21.91	6.09	14.88	9.10
	AT4G10250	HSP22.0	1.00	639.22	1.00	1.00	422.32	12,197.30	29,742.20	14,572.00	31,081.90	27,217.10
	AT4G27670	HSP21	1.00	0.75	0.87	3.15	2.47	1.53	3.16	2.03	7.44	4.17
	AT5G12030	HSP17.6A	1.00	2.67	1.14	2.01	3.52	54.30	99.39	58.48	114.72	95.71
	AT5G12020	HSP17.6II	1.00	1.77	1.68	1.12	1.82	6.07	12.77	10.21	14.79	10.33
	AT2G29500	HSP17.6B	1.00	4.32	1.41	1.78	3.51	10.18	16.52	14.67	23.52	16.23
ABA- related genes	AT1G70800	CAR6	1.00	2,468.02	2,155.69	1.00	1,630.66	4,204.89	2,609.99	2,009.45	1,363.79	1,251.14
	AT1G70910	DEP	1.00	2,565.57	1.00	1,080.24	847.51	3,496.78	2,713.02	1.00	2,835.23	1,300.46
	AT2G40220	ABI4	1.00	936.68	2,181.63	394.39	309.42	638.33	3,962.04	1.00	517.56	1.00
	AT5G52310	RD29A	1.00	3.54	1.01	2.84	4.16	4.12	13.88	4.31	8.49	4.81

*RNA sequencing was used to analyze transcriptomes of 7-day-old plants cultivated at 22°C (con) or treated with heat at 44°C (H). The gene expression levels were calculated as FPKM (Fragments Per Kilobase of transcript per Million mapped reads). Their ratios relative to those of WT under 22°C are shown as the relative expression levels in **(b)***.

## Discussion

Heat is one of the most serious stresses affecting plant growth, development, and crop yields. sRNAs play important roles in heat stress. In *Arabidopsis*, the intronic miR400 co-transcribed with its host gene (*At1g32583*) was downregulated after heat treatment (Yan et al., 2012). Heat stress also induced the expression of heat-induced trans-acting siRNA precursor 1 (TAS1) target 1 (*HTT1*) and *HTT2* through the reduced expression of TAS1-siRNAs (Li et al., 2014). In wheat, high-throughput sequencing verified that high temperature decreases the expression of Ta-miR172 and increases the expression of Ta-miR156, Ta-miR159, Ta-miR160, Ta-miR166, Ta-miR168, Ta-miR169, Ta-miR393, and Ta-miR827 (Xin et al., 2010). In heat stress, the induction of barley miR160 expression correlates with downregulation of *ARF17* and *ARF13* (Kruszka et al., 2014). The promoter region of miR160 precursor gene in cassava (Pinweha et al., 2015) and tomato (Lin et al., 2015) contained HSEs. In this study, gene expression analysis also revealed miR160 and it precursors were induced in *Arabidopsis* after exposure to heat treatment (Figures 1A,B). Under the same condition, three miR160 targets, namely *ARF10, ARF16*, and *ARF17*, were significantly repressed (Figure 1C). Therefore, the interaction of miR160/*ARF10, 16*, and *17* might play a role in the development of plants in response to heat stress, which also involves regulation of the gene expression of HSPs.

Several molecular mechanisms and signals play important roles in the thermotolerance of plants. Under heat stress, auxin signaling moderated anther fertility in *Arabidopsis*, barley, and cotton (Sakata et al., 2010; Oshino et al., 2011; Ding et al., 2017). In cotton, miR160 cleaved *ARF10, ARF16*, and *ARF17* mRNA to mediate auxin signaling under high temperate (Ding et al., 2017). The expression of *ARF10, ARF16*, and *ARF17* was lower and higher in 160OE and MIM160 plants than in WT under heat stress (Figures 2A,B), respectively, indicating the activity of miR160 was capable of regulating these *ARFs* expression. Thus, miR160 might directly repress *ARF10, ARF16*, and *AR*F17 in heat stress. However, the inhibition of miR160 activity in MIMI160 plants could not completely restore these *ARFs* expression under heat stress (Figure 2B), indicating factors other than miR160 may be involved in *ARFs* repression. Auxin can regulate the expression of *ARF10* and *ARF16* through miR160-independent mechanism (Wang et al., 2005). Auxin contents were decreased in barley and *Arabidopsis* treated with heat stress (Oshino et al., 2007; Sakata et al., 2010). *ARF10, ARF16*, and *ARF17* were significantly repressed by heat stress (Figure 1). Taken together, these results indicated that high temperature decreased auxin to repress *ARFs*, and that the repression of *ARFs* was directly or indirectly regulated by miR160 and auxin in plants under heat stress.

Germination capacity and germination progression of seeds were affected by heat (Silva-Correia et al., 2014). The germination percentages were better for 160OE than WT seeds, under heat stress, while seeds of MIM160 were sensitive to heat (Figure 3B). In addition, loss of function mutants *arf10-1, arf16-3*, and *arf17-2* plants increased the seed germination percentages after heat treatment (Figure 7A). These results indicated that *ARF10, ARF16*, and *ARF17* were repressed by miR160 to elevate seed germination in heat stress. For seed germination, the balance between gibberellin and ABA is an essential factor in *Arabidopsis* (Bewley, 1997; Seo et al., 2006). ABA contents of *Arabidopsis* seeds are increased when treated with high temperature (Toh et al., 2008). During seed germination, miR160 is involved in the regulation of auxin-ABA crosstalk and reduces the ABA effect (Liu et al., 2007). Conversely, seeds of transgenic plants overexpressing miR160-resistant form of *ARF10* are hypersensitive to ABA (Liu et al., 2007). *ABI3* is considered to be a major downstream component of ABA signaling (Bentsink and Koornneef, 2008). ARF10 and ARF16 regulate *ABI3* expression to induce seed dormancy (Liu et al., 2013). UGT75D1 decreased ARF16-ABI3 signaling to mediate germination (Zhang et al., 2016). These studies indicated that miR160 target genes, namely *ARF10* and *ARF16*, negatively affected seed germination. The expression of miR160 targets, *ARF10, ARF16*, and *ARF17*, was repressed and elevated in 160OE and MIM160 plants, respectively (Figures 2A,B). Taken together, miR160-induced repression of *ARF10, ARF16*, and *ARF17* expression might decrease ABA-mediated inhibition of seed germination under heat stress.

The miR160/*ARF10, 16, 17* mechanism participates in leaf development (Ren and Tang, 2012), root formation (Gutierrez et al., 2009; Liang et al., 2012), and cell differentiation (Qiao et al., 2012). Several sRNAs involved in plant development regulate the responses to abiotic stress. When *Arabidopsis* is grown in nitrogen-deficient soil, miR160 is induced to control lateral root formation (Liang et al., 2012). In response to heat stress, miR156 modulates plant development in *Arabidopsis*, through regulating *SPL* (Stief et al., 2014). The hypocotyl elongation assay was used to screen genes involved in thermotolerance (Hong and Vierling, 2000). In heat stress, the hypocotyl elongates through modulating auxin contents (Sun et al., 2012). Auxin is an important factor involved in hypocotyl growth. High temperature induces *PIF4* expression to promote *YUCCA8* expression, further elevating auxin biosynthesis (Sun et al., 2012). The interaction of miR160 and its targets ARFs plays important roles in auxin signaling of plant growth (Liu et al., 2007). Upon heat stress, 160OE and MIM160 plants could elevate and reduce hypocotyl elongation lengths, respectively (Figure 5B). Furthermore, mutants of *ARF16* and *ARF17* increase hypocotyl elongation lengths (Figure 7C). In *Arabidopsis, ARF17* negatively alters hypocotyl growth (Mallory et al., 2005). With auxin treatment, the hypocotyl growth of transgenic plants overexpressing miR160-resistant *ARF17* occurs slowly compared to those without auxin treatment (Mallory et al., 2005). All these results indicated that miR160 regulated hypocotyl growth through auxin signaling.

In addition to plant development, interestingly, the gene expression levels of several HSPs were increased in 160OE, *arf10-1, arf16-3*, or *arf17-2* plants, as detected by RNA sequencing analyses (Table 1). HSFs and HSPs are considered to be the central components involved in heat tolerance of plants (Nover and Scharf, 1997; Kotak et al., 2007). Under heat stress, miRNA also affects *HSF*s and *HSP*s expression to regulate the abilities of thermotolerance. The miR156/SPL mechanism regulates heat memory via regulating the expression of *HSA32, HSFA2, HSP17.6A*, and *HSP22* (Stief et al., 2014). *HTT1*, which is regulated by miR173/TAS1, acts as a cofactor in the Hsp70-14-NF-YC2 complex to enhance thermotolerance (Li et al., 2014). The miR398/*CSD* mechanism can regulate ROS contents to mediate *HSF* expression in *Arabidopsis* in response to heat (Guan et al., 2013). Under heat stress, overexpression of miR160 in *Arabidopsis* enhanced the expression of *HSP21, HSP17.6A*, and *HSP17.6II* compared to those of WT (Figure 8A, Table 1). Conversely, the expression of *HSP101, HSP21, HSP17.6A*, and *HSP17.6II* was decreased in plants when miR160 expression was reduced (Figure 8B). In *arf10-1, arf16-3*, or *arf17-2* plants, the expression of *HSP101, HSP21, HSP17.6A*, and *HSP17.6II* was also altered after heat treatment (Table 1 and Supplemental Figure S5).

These results indicated that miR160 might regulate the gene expression of *HSP*s during heat stress. HSPs are involved in the stabilization of proteins denatured by stress and the maintenance of accuracy in early protein folding (Gustavsson et al., 2002). *HSP101* plays a key role in thermotolerance in *Arabidopsis* and affects hypocotyl growth and seed germination after heat treatment (Queitsch et al., 2000). Under heat stress, *HSP21* participates in protecting the thermolabile photosystem II (Wang and Luthe, 2003; Neta-Sharir et al., 2005) and also maintains the plastid-encoded RNA polymerase to regulate chloroplast development (Zhong L. et al., 2013). Furthermore, HSP21 acts as an essential factor in the early development of seedlings under heat stress (Zhong L. et al., 2013). In *Arabidopsis*, salicylic acid regulates *HSP17.6* to promote basal thermotolerance (Clarke et al., 2004). *HSP101* (Wu et al., 2013), *HSP21* (Shahnejat-Bushehri et al., 2012), and *HSP17.6A* (Stief et al., 2014) are involved in thermomemory and enhance heat tolerance. Moreover, *HSP101, HSP21, HSP17.6A*, and *HSP17.6II* protect plant cells from heat-induced programmed cell death (Rikhvanov et al., 2007). 160OE and MIM160 plants showed heat tolerance and heat sensitivity, respectively, via regulating the expression of *ARF10, 16*, and *17*. Thus, miR160 might repress its target gene expression to regulate *HSP* genes and control thermotolerance of plants.

**Figure 9:**
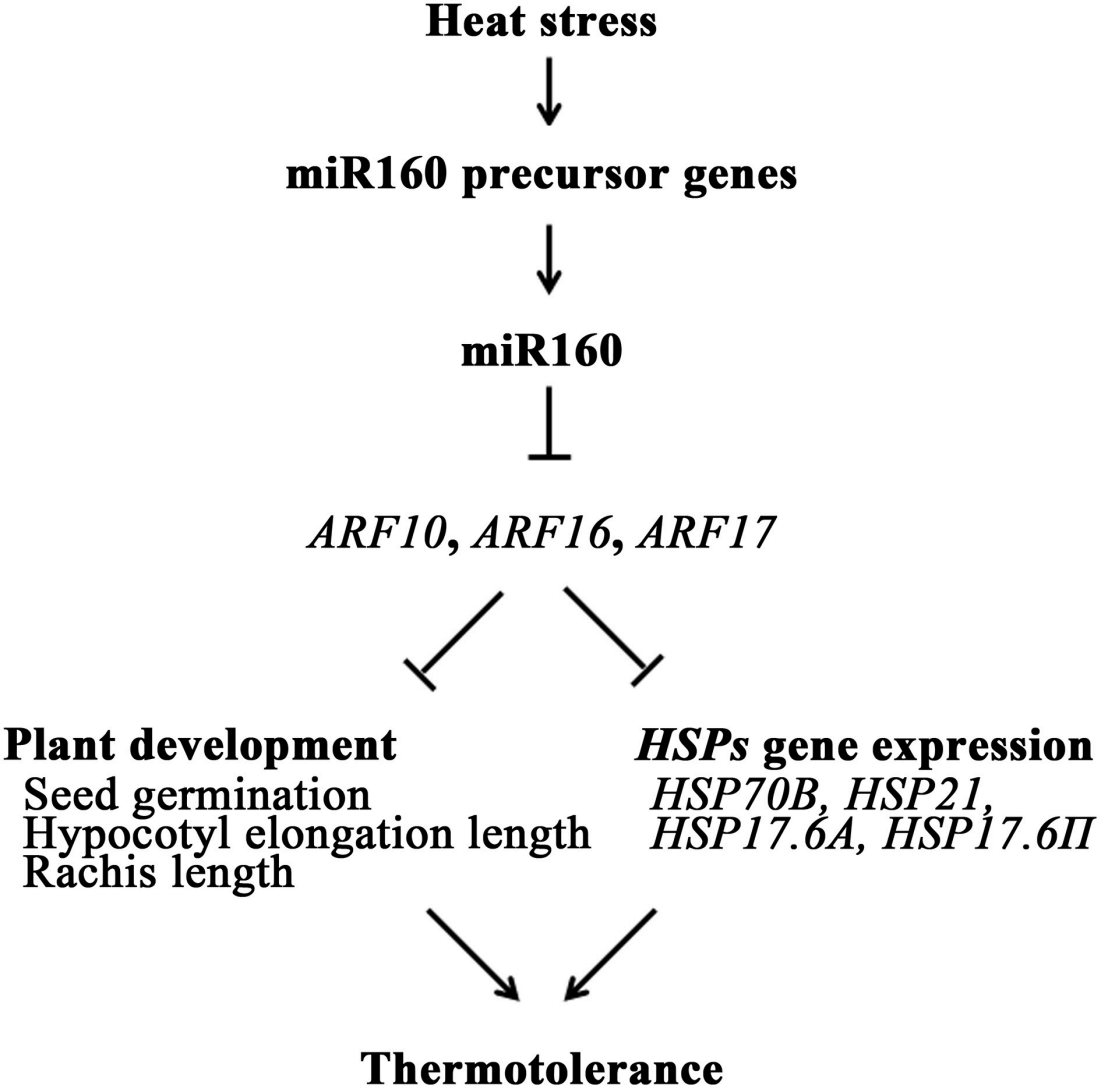
Schematic of miR160-mediated response in Arabidopsis upon heat stress. Heat stress could induce miR160 precursor genes, *pre-miR160a, pre-miR160b*, and *pre-miR160c*, to increase mature miR160. Then, miR160's targets, *ARF10, ARF16*, and *ARF17*, were repressed by miR160, and regulate the seed germination, hypocotyl growth, and rachis growth. In addition, several *HSP* genes were activated by miR160 and regulated by *ARF10, ARF16*, and *ARF17*. Thus, thermotolerance of plants was enhanced.

Conclusively, miR160 was induced in heat stress to repress the expression of its targets, *ARF10, ARF16*, and *ARF17*, which regulated seed germination, hypocotyl growth, and rachis growth of *Arabidopsis*. In addition, the expression levels of *HSP* genes were regulated by *ARF10, ARF16*, and *ARF17* (Table 1b). These miR160-induced regulation mechanisms could elevate the thermotolerance of plants (Figure 9). Therefore, this study advances the understanding of miR160 functions in heat stress. The miR160/ARFs mechanism not only affects plant development but also regulates the gene expression of HSPs.

## Significance statement

The induction of microRNA160 (miR160) repressed *ARF10, 16*, and *17* to affect plant development and *HSPs* gene expression, resulting in elevating thermotolerance of plant.

## Author contributions

J-SL and S-TJ designed the research. C-CK performed most of the experiments, and J-SL, I-CY, W-AT, Y-HS, C-CL, Yi-CL, Yu-CL, Y-WK, Y-CK, and H-ML provided substantial help in specific experiments. J-SL and W-AT created transgenic plants overexpressing miR160 and target mimic. J-SL, H-ML, and STJ wrote the article. All authors read and approved the final manuscript.

### Conflict of interest statement

The authors declare that the research was conducted in the absence of any commercial or financial relationships that could be construed as a potential conflict of interest.
