# Supplementary material for: MicroRNA160 Modulates Plant Development and Heat Shock Protein Gene Expression to Mediate Heat Tolerance in Arabidopsis
Source: Front Plant Sci. 2018 Feb 1;9:68. doi: 10.3389/fpls.2018.00068 (PMC5799662; doi:10.3389/fpls.2018.00068)
Supplement: Supplementary file 1 [file Presentation1.PDF]

Supplemental Data

Supplemental Table S1. Primers used in this study

BamHI-premiR160a F	GGATCCAAATCGTCATTTAAGGCTTCAAG
SacI-premiR160a R	GAGCTCGCATATGATGGATTCGTGAGGTA
mimic160 fragment	GAGGGA ACCGAAGCTTGGCATACAGGCTAGAGCCAGGCATTT CTAGAGGGAGATAAGAGC
BamHI-mimic F	GGATCCGAGGGA ACCGAAGCT
SacI-mimic R	GAGCTCGCTCTTATCTCCCTCTAGAAA
T25VN	TTTTTTTTTTTTTTTTTTTTTTTTTVN
pre-miR160a F	TGCCTGGCTCCCTGTATG
pre-miR160a R	CCATCCACGGAGGTCATC
pre-miR160b F	TCCCTGTATGCCACAAGAAA
pre-miR160b R	CGCCACTAGTGATCGATTTT G
pre-miR160c F	GCTCCCTGTATGCCACGAGT
pre-miR160c R	CAGCCGATTTTAAAACCAAAA
ARF10 F	TTCCGCAGCCATTTGAGTTTC
ARF10 R	AGATGGTGATCCGAAGAGTTGT
ARF10 F2	TAGAAGAGAGGTCGGATTTGTTGACTCAT
ARF10 R2	CCACTGCTTATTCCTCAATATGCTTCAAG
ARF16 F	CCCGTTAAGCTCTGTTCTGGAC
ARF16 R	TTCGGAGACCGAGAGAAGGAG
ARF16 F2	ATTAGCGGAAGACCGTTTGAGGTTGTGTA
ARF16 R2	ATTGTTGTAATCTGGATGCTGAGGTAGCC
ARF17 F	CAGCAGCACCTGATCCAAGTC

ARF17 R	GACTGCCAAAGTTCATCATCTCAG
ARF17 F2	AGTGAAAGAAGTGGTGGATCTTTGCTGGA
ARF17 R2	AGCGAGATGTTCATCAAATGTGTCTGAGA
HSP101 F	GGCTTGTGCGAATGTGAGAGTCC
HSP101 R	GAGGCTGAAGCTTGTCTCTCAGGTC
HSP70B F	AGCTATTGGTATCGATCTCGGCACTAC
HSP70B R	AAGCCTCAGCGACTTCCTTCATCTTCAC
HSP21 F	TGTCACCAATGAGGACGATGCGA
HSP21 R	TGATGTCCCACGGTGCACGA
HSP17.6A F	TGTGAGGATGGAGAGGAGGATGGG
HSP17.6A R	ACACCGTCATTACAAGCCGCAGAG
HSP17.6II F	ACAACAACGAGAAGACCCGCAACA
HSP17.6II R	CGTCAGCAGGTGTAGCAGCCATT
Actin F	ACCTTGCTGGACGTGACCTTACTGAT
Actin R	GTTGTCTCGTGGATTCCAGCAGCTT
T3 top strand	ATGAATTAACCCTCACTAAAG
T3-miR160	TGCCTGGCTCCCTGTATGCCACTTTAGTGAGGGTTAATTCAT
5.8S rRNA F	AAATACGACTCTCGGCAACGG
5.8S rRNA R	AACTTGCGTTCAAAGACTCGAT

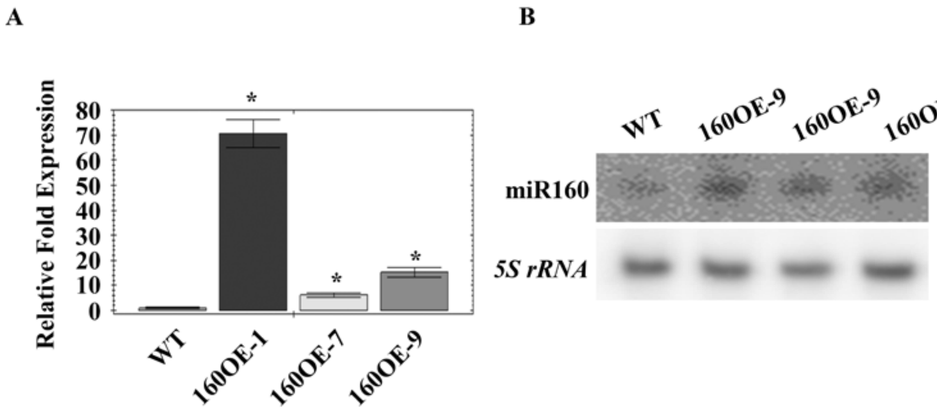

Supplemental Figure S1. Expression of miR160 and its precursor in WT and 160OE plants under heat stress

The 7-day-old WT and 160OE seedlings were treated with heat stress at 44°C for 1 hour. The total RNAs from these seedlings were extracted and analyzed. The expression of miR160 precursor (A) was analyzed using quantitative RT-PCR, and normalized to the levels of *Actin* expression. The mature form of miR160 (B) was also detected by northern blotting. *5S rRNA* were used as loading controls.

Statistic differences between WT and transgenic plants are marked with *star* according to hypothesis testing (*: $P < 0.05$).

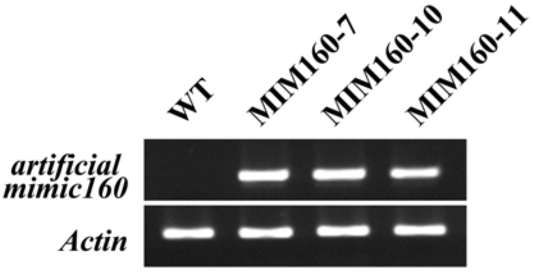

Supplemental Figure S2. Expression of artificial miR160 target mimic inhibitor in WT and MIM160 plants

The total RNAs from 7-day-old WT and MIM160 seedlings were extracted and analyzed. The expression of artificial miR160 target mimic inhibitor (*mimic160*) was analyzed using semi-quantitative RT-PCR. The expression levels of *Actin* were controls for comparison.

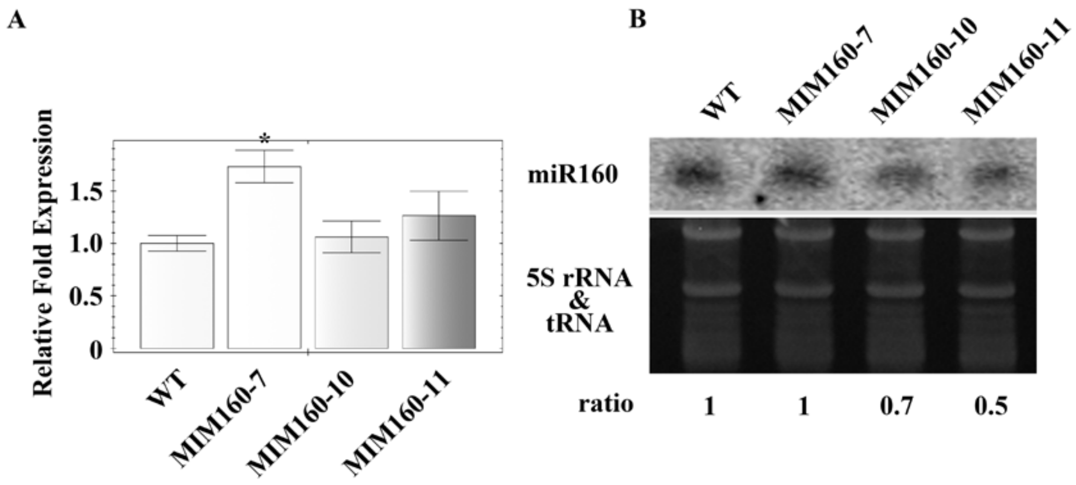

Supplemental Figure S3. Expression of miR160 and its precursor in WT and MIM160 plants under heat stress

The 7-day-old WT and MIM160 seedlings were treated with heat stress at 44°C for 25 min. The total RNAs from these seedlings were extracted and analyzed. The expression of miR160 precursor (A) was analyzed using quantitative RT-PCR, and normalized to the levels of *Actin* expression The mature form of miR160 (B) was also detected by northern blotting. *5S rRNA* and tRNA stainings were used as loading controls. Statistic differences between WT and transgenic plants are marked with *star* according to hypothesis testing (*: P < 0.05).

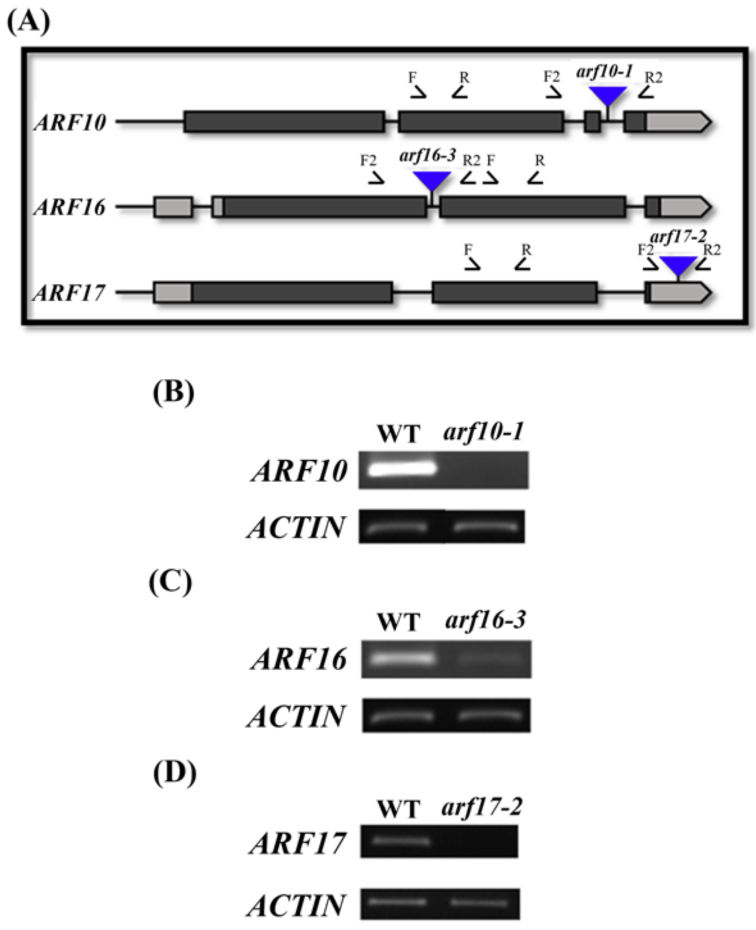

Supplemental Figure S4. Expression of the T-DNA insertion mutants *arf10-1*, *arf16-3*, and *arf17-2* under heat stress

The T-DNA insertion sites of *arf10-1*, *arf16-3*, and *arf17-2* were presented (A). The inverted triangles indicate the T-DNA insertion sites. Arrows labeled F and R were used to detect the gene expression of *ARF10*, *ARF16*, and *ARF17*, while those labeled F2 and R2 were for identifying T-DNA insertion lines. The total RNAs from 7-day-old WT and the T-DNA insertion mutants *arf10-1* (B), *arf16-3* (C), and *arf17-2* (D) seedlings were extracted and analyzed by semi-quantitative RT-PCR with primer pairs ARF10 F2/ARF10 R2, ARF16 F2/ARF16 R2, and ARF17 F2/ARF17 R2 (Supplemental Table S1), respectively. Expression levels of *Actin* act as internal controls for comparison.

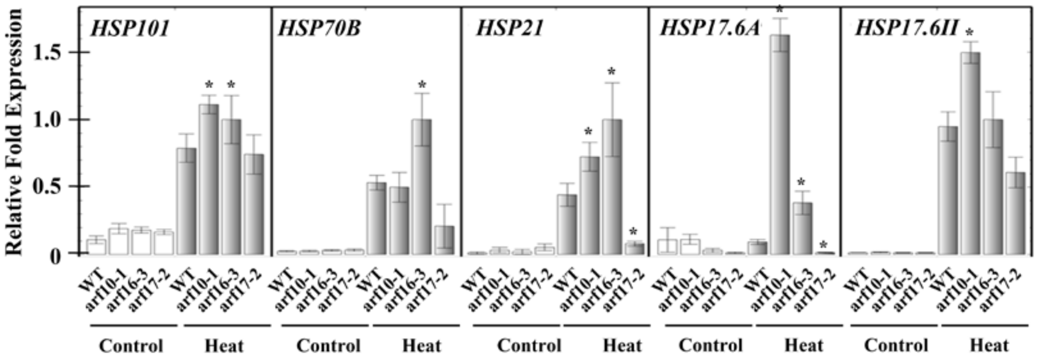

Supplemental Fig. S5. Expression of *HSP*s in WT, *arf10-1*, a*rf16-3*, and *arf17-2* plants under heat stress

The 7-day-old WT, *arf10-1,* a*rf16-3,* and *arf17-2* seedlings were treated with heat stress at 44°C for 25 min, respectively. Seedlings without heat stress were included for comparison. The total RNAs from these seedlings were extracted and analyzed by RT-PCR. The expression of *HSP101*, *HSP21*, *HSP18*, *HSP17.6A*, and *HSP17.6II* was analyzed using quantitative RT-PCR, and normalized to the levels of *Actin* expression. Their ratios relative to those of heat-treated WT are shown as the relative expression levels. Treatment conditions are shown on the left panels. Statistic differences between WT and transgenic plants are marked with *star* according to hypothesis testing (*: $P < 0.05$).
